# ITRAQ-Based Quantitative Proteomics Reveals the Proteome Profiles of Primary Duck Embryo Fibroblast Cells Infected with Duck Tembusu Virus

**DOI:** 10.1155/2019/1582709

**Published:** 2019-01-27

**Authors:** Feng Hu, Yufeng Li, Kexiang Yu, Bing Huang, Xiuli Ma, Cunxia Liu, Xiaozhen Guo, Minxun Song, Jiaqiang Wu

**Affiliations:** ^1^Institute of Poultry Science, Shandong Academy of Agricultural Sciences/Shandong Provincial Key Laboratory of Immunity and Diagnosis of Poultry Diseases, No. 1 Jiaoxiao Road, Jinan, Shandong 250023, China; ^2^College of Life Sciences, Shandong Normal University, 88 East Culture Road, Jinan, Shandong 250014, China

## Abstract

Outbreaks of duck Tembusu virus (DTMUV) have caused substantial economic losses in the major duck-producing regions of China since 2010. To improve our understanding of the host cellular responses to virus infection and the pathogenesis of DTMUV infection, we applied isobaric tags for relative and absolute quantification (iTRAQ) labeling coupled with multidimensional liquid chromatography-tandem mass spectrometry to detect the protein changes in duck embryo fibroblast cells (DEFs) infected and mock-infected with DTMUV. In total, 434 cellular proteins were differentially expressed, among which 116, 76, and 339 proteins were differentially expressed in the DTMUV-infected DEFs at 12, 24, and 42 hours postinfection, respectively. The Gene Ontology analysis indicated that the biological processes of the differentially expressed proteins were primarily related to cellular processes, metabolic processes, biological regulation, response to stimulus, and cellular organismal processes and that the molecular functions in which the differentially expressed proteins were mainly involved were binding and catalytic activity. Some selected proteins that were found to be differentially expressed in DTMUV-infected DEFs were further confirmed by real-time PCR. The results of this study provide valuable insight into DTMUV-host interactions. This could lead to a better understanding of DTMUV infection mechanisms.

## 1. Introduction

Duck Tembusu virus (DTMUV), which belongs to the* Flavivirus* genus, is the causative agent of egg-drop syndrome in multiple avian hosts, including ducks, geese, chickens, pigeons, and house sparrows [1–4]. Outbreaks of DTMUV have caused large economic losses in China since 2010. Moreover, DTMUV can also replicate in mice, with high neurovirulence and age-dependent neuroinvasiveness, which poses a potential public health concern [5–7]. Infection of DTMUV mainly causes a decline in egg production, acute anorexia, antisocial behavior, rhinorrhea, diarrhea, ataxia, and paralysis [4]. Recently, diagnostic methods and vaccines for DTMUV have been successfully developed and already used in clinical production, which provides a method for better prevention and treatment of the disease [8–13]. In addition, many host factors are likely to play critical roles in the DTMUV life cycle including glucose-regulated protein 78, heat shock protein A9, proinflammatory cytokines, and antiviral proteins [14–18]. However, current knowledge of proteomic information about duck cell line responses to DTMUV infection is still limited.

Knowledge of the virus-host interaction is critical for understanding the pathogenesis of viral infection. Currently, proteomic approaches have been used for studying the viral pathogenesis [19, 20]. Han et al. [21] identified 131 host proteins that were altered in duck ovarian follicles following DTMUV infection using a label-free quantitative proteomic method. Isobaric tags for relative and absolute quantification (iTRAQ) as a high-throughput proteomics approach are useful for the analysis of infection-associated proteins of pathogens [22–24]. Sun et al. [25] identified 192 significantly expressed host proteins in a DTMUV-infected baby hamster kidney cell line using the iTRAQ approach.

We carried out our research on the basis of these previous studies. In the current study, iTRAQ combined with tandem mass spectrometry (LC-MS/MS) was used to conduct proteomic analysis of DEFs infected with DTMUV to explore the possible mechanisms of virus infection. A total of 116 significant and differentially expressed host proteins were identified at 12 hours postinfection (hpi), 76 at 24 hpi, and 339 at 42 hpi. Analysis and functional studies of these altered expression proteins might provide fundamental information for the study of virus-host interactions and the molecular basis underlying DTMUV pathogenesis.

## 2. Materials and Methods

### 2.1. Cells and Virus

The 10-day-old specific-pathogen-free (SPF) duck embryos were provided by the Institute of Poultry Science, Shandong Academy of Agricultural Sciences, and were used to prepare DEFs. DEFs were maintained in DMEM (Gibco, USA) supplemented with 10% fetal bovine serum (Gibco, USA) at 37°C in a 5% CO_2_ atmosphere. The DTMUV BZ-2010 strain (GenBank Accession No. KC990540) was propagated in DEFs to a titer of 10^6.0^ TCID_50_/ mL and maintained in our laboratory.

### 2.2. Virus Inoculation

DEFs were cultured to approximately 80% confluence and then inoculated with 10^2.0^ TCID_50_ of DTMUV. After a 2 h exposure to the virus, the cells were washed three times with ice-cold PBS and cultured in DMEM supplemented with 1% fetal bovine serum. Uninfected DEFs served as mock-infected cells. The infected and uninfected DEFs were harvested at 12, 24, and 42 hpi, respectively. DTMUV infection was verified by observation of the cytopathic effect (CPE), virus titers determination, and virus genome copy number.

### 2.3. Sample Preparation, Protein Digestion, Desalting, and iTRAQ Labeling

The infected and uninfected DEFs were washed twice with ice-cold PBS, collected by cell scraping, and centrifuged at 300 × g for 10 min. Three biological replicates of the DTMUV- or mock-infected groups were well mixed when collecting the samples. The collected cells were lysed in 200 *μ*L of dissolution buffer (7 M urea, 2 M thiourea, 4% SDS, 40 mM Tris-HCl, pH 8.5, 1mM PMSF) and broken by sonication for 15 min. Then, the mixtures were centrifuged at 15,000 × g for 20 min. The proteins were extracted using cold acetone, dried, and then dissolved in triethylammonium bicarbonate buffer (TEAB, pH8.0). The extracted peptides were reduced with DTT and alkylated with iodoacetamide (IAM), and then the concentration was determined using the Bradford protein assay [26]. For each sample, 100 *μ*g of protein was dissolved in TEAB buffer and then trypsin-digested. After being purified on a Strata–X C18 column (Phenomenex, Torrance, CA, USA), the eluted peptides were labeled with an iTRAQ Reagent-8 plex Multiplex Kit (AB Sciex U.K., Ltd.) according to the manufacturer's instructions.

### 2.4. LC-MS/MS Analysis

All of the iTRAQ labeled peptides were mixed and then fractionated by a high-performance liquid chromatography (HPLC) system (Thermo DINOEX Ultimate 3000 BioRS) using a Durashell C18 column (5 *μ*m, 100 Å, 4.6 × 250 mm; Agela Technologies, Tianjin, China). The LC-MS/MS analysis was performed as described previously by using an AB SCIEX nano LC-MS/MS (TripleTOF 5600 plus, AB SCIEX, USA) system [27]. Each fraction was dissolved in aqueous solution containing 0.1% FA and 3% ACN. The mobile phases were composed of solvent buffer A (5% ACN, 0.1% FA) and buffer B (95% ACN, 0.1% FA). The gradient run was from 5 to 50% buffer B for 70 min at 300 nL/min, maintained at 80% buffer B for 15 min, and finally returned to 5% buffer B for 5 min. During data acquisition, MS spectra were acquired in the range 350-1,500 m/z for 250 ms. The 20 most MS/MS (resolution ≥15000) were selected in the range 50-2,000 m/z from each MS spectrum with 100 ms. The dynamic exclusion of precursor ions was fixed for 20s.

### 2.5. Data Analysis

Protein identification was performed with the ProteinPilot™ software (Version: 4.5; Applied Biosystems) using Paragon™ Algorithm as database search engine. The MS data were searched against the Uniprot Anas database (34035 sequences, downloaded on July 14, 2017). The parameters were set as follows: the instrument was TripleTOF 5600, iTRAQ quantification, cysteine modified with iodoacetamide, biological modifications were selected as ID focus, trypsin digestion, the Quantitate, Bias Correction and Background Correction was checked for protein quantification and normalization. For false discovery rate (FDR) analysis, an automatic decoy database search strategy was employed to estimate FDR using the PSPEP (Proteomics System Performance Evaluation Pipeline Software, integrated in the ProteinPilot Software) algorithm. For quantification, proteins with at least one unique peptide and an unused value greater than 1.3 were considered for further analysis. Proteins with a fold change >1.5 or <0.67 and a p-value <0.05 were considered to be significantly different expressions.

### 2.6. Bioinformatic Analysis

Functional protein analyses were extracted using the AmiGO tool in the Gene Ontology platform (http://geneontology.org). Pathway analyses were extracted using the search pathway tool in the KEGG Mapper platform (http://www.genome.jp/kegg/mapper.html).

### 2.7. RNA Extraction and Real-Time PCR Analysis

Total cellular RNA was extracted from the DTMUV-infected and mock-infected DEFs using AxyPrep Multisource Total RNA Miniprep Kit (Axygen, CA, USA) according to the manufacturer's instructions, and the cDNA was synthesized using PrimeScript™ RT Master Mix (Takara, Dalian, China). The primers (Table 1) were synthesized by TsingKe Biotechnology Company (Beijing, China). Quantitative real-time PCR was performed using the Roche LightCycler 96 real-time PCR system. In order to analyze the identified proteins at the transcriptional level, relative quantitative real-time PCR was performed in a 20 *μ*L volume including 10 *μ*L SYBR® Premix Ex Taq II, 1 *μ*L each primer (10 pM), and cDNA template. Cycle conditions were as follows: one cycle at 95°C for 30 s, and then 40 cycles at 95°C for 5 s, 56°C for 10 s, and 72°C for 10 s, and melting curves were obtained. In order to monitor DTMUV replication kinetics, absolute quantitative real-time PCR was carried out. The fragment targeting DTMUV capsid protein gene was amplified by PCR using pair primers (Table 1) and cloned into pMD18-T vector (TaKaRa, Dalian, China) to construct the standard plasmid DNA, namely, pMD18-C. Then, the concentration of the plasmid standard was quantified using optical density determination at 260 nm, and the serial dilutions of plasmid standard were used to establish the standard curve. Absolute quantitative real-time PCR was performed in a 20 *μ*L volume containing cDNA template, 10 *μ*L SYBR® Premix Ex Taq II, 1 *μ*L each primer (10 pM). Cycle conditions were as follows: one cycle at 95°C for 30 s, and then 40 cycles at 95°C for 15 s, 55°C for 15 s, and 72°C for 20 s, and melting curves were obtained. Each cDNA sample was amplified in triplicate. The data analysis was performed using the Roche LightCycler 96 real-time PCR system software. Relative transcript levels were calculated using the ΔΔCt method as specified by the manufacturer. *β*-actin was employed as an internal reference gene. The statistical analyses were performed using the GraphPad Prism 6.0 software. Student's* t*-test and one-way ANOVA were used to evaluate the significance of genomic RNA copies, and a value of P <0.05 was considered significant.

## 3. Results

### 3.1. Confirmation of DTMUV Infection in DEFs

**Figure 1:**
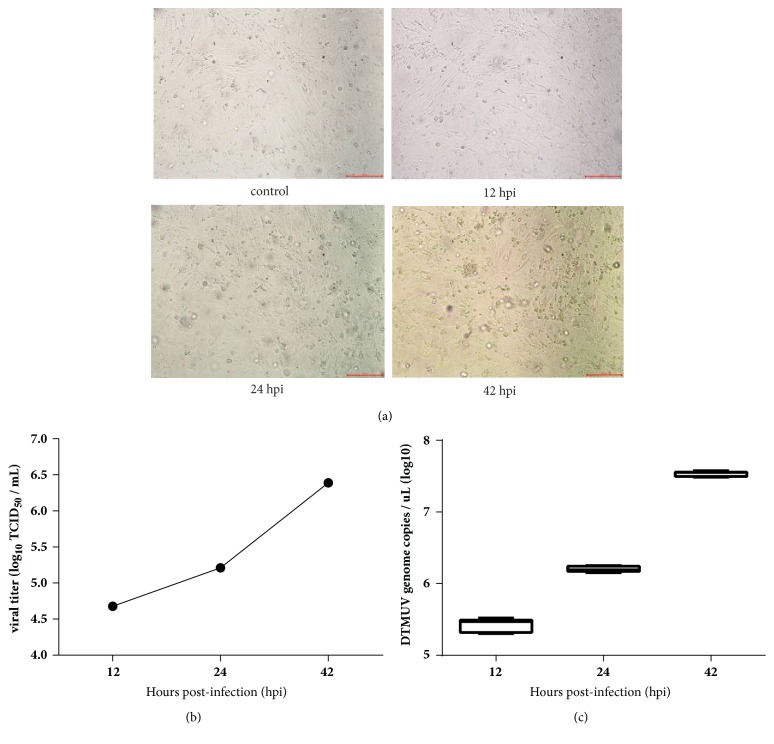
DTMUV infection in DEFs. (a) The cytopathic effects (CPE) of DEFs at 12, 24, and 42 h after infection, and mock-infected cells as control. (b) Virus titers determination of DTMUV in DEFs at 12, 24, and 42 hpi. (c) DTMUV genome load in infected DEFs. Cells were infected with the BZ-2010 strain of DTMUV and collected at 12, 24, and 42 hpi. DTMUV genome copy numbers were quantitated using absolute quantitative real-time PCR. Error bars represent the standard error of the mean from three independent experiments.

Successful DTMUV infection was verified by observation of the cytopathic effect (CPE), virus titers determination, and virus genome copy number. The results were presented in Figure 1. CPE was not visible at 12 hpi and 24 hpi, and apparent CPE could be observed at 42 hpi (Figure 1(a)). As shown in Figure 1(b), the viral titer reached 4.68, 5.21, and 6.39 log_10_TCID_50_/mL at 12, 24, and 42 hpi, respectively. As shown in Figure 1(c), in the DTMUV-infected group, the levels of the viral genome were detected at 12, 24, and 42 hpi, indicating the development of persistent infection. Virus was not detected in DEFs in the control group.

### 3.2. Protein Profile Obtained by iTRAQ LC-MS/MS Analysis

**Figure 2:**
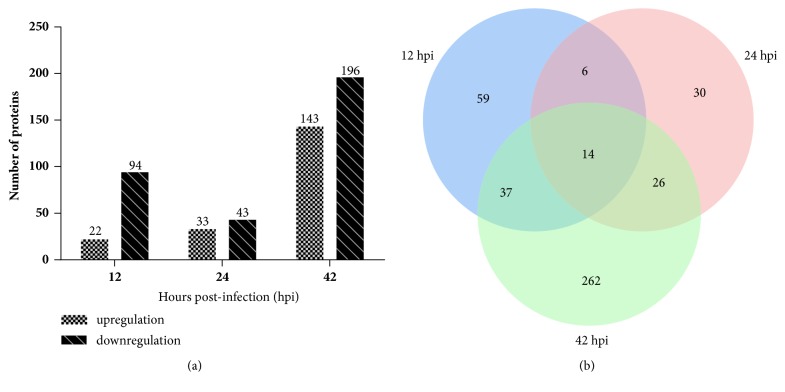
Numbers of differentially expressed proteins from DEFs infected with DTMUV. (a) Number of significant and differentially expressed proteins during infection with DTMUV relative to mock-infected DEFs (p <0.05, fold change >1.5 or <0.67). (b) Venn diagram displays the distribution of differentially expressed proteins during infection with DTMUV.

A total of 4283 proteins, including 20005 peptides, were identified in the DTMUV-infected and mock-infected groups (data not shown). A total of 434 proteins displayed significant and differentially expressed levels upon infection, among which 389 were known proteins, and 45 were uncharacterized proteins (SI Table 1). Among these, 116, 76, and 339 proteins were differentially expressed relative to uninfected DEFs at 12, 24, and 42 hpi, respectively. Of the 116 differentially expressed proteins at 12 hpi, 22 proteins were upregulated and 94 proteins were downregulated. Of the 76 differentially expressed proteins at 24 hpi, 33 proteins were upregulated and 43 proteins were downregulated. Of the 339 differentially expressed proteins at 42 hpi, 143 proteins were upregulated and 196 proteins were downregulated (Figure 2(a)). In addition, a Venn diagram analysis revealed that 14 significant and differentially expressed proteins were commonly represented at all times postinfection (Figure 2(b)).

### 3.3. GO Analysis of Differentially Expressed Proteins

**Figure 3:**
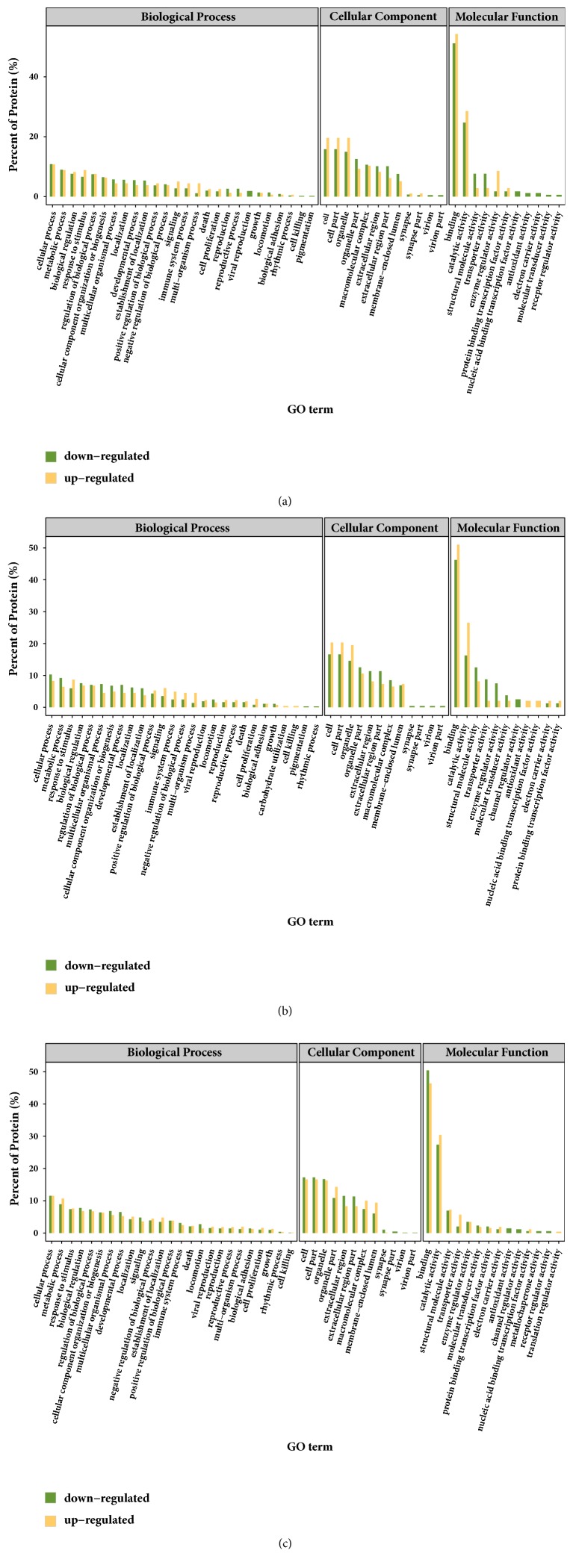
GO analysis of the differently expressed proteins based on biological process, cellular component, and molecular function at 12 hpi (a), 24 hpi (b), and 42 hpi (c).

A total of 434 significantly expressed proteins were categorized according to the GO molecular functional groups: biological processes, cellular components, and molecular functions (Figure 3). The biological process annotation revealed that these significant and differentially expressed proteins were primarily involved in cellular process (such as lactate biosynthetic process, pyruvate biosynthetic process, and NADPH regeneration), metabolic processes (such as protein folding, ribose phosphate biosynthetic process, gluconeogenesis, and pentose-phosphate shunt), biological regulation (such as protein stabilization and protein destabilization), response to stimulus, developmental processes, and cellular component organization or biogenesis. The cellular component annotation revealed that these differentially expressed proteins were mainly involved in the cell (such as Schmidt-Lanterman cleft and myelin sheath), cell part (such as cytoplasmic vesicle and intracellular organelle), organelle (such as melanosome), and extracellular region (such as extracellular vesicular exosome). The molecular function annotation revealed that the differentially expressed proteins were mainly distributed among two molecular function groups: binding (such as cyclosporin A binding, macrolide binding, cocaine binding, and enzyme binding) and catalytic activity (such as GTPase regulator activity).

### 3.4. KEGG Pathway Analysis of Differentially Expressed Proteins

**Figure 4:**
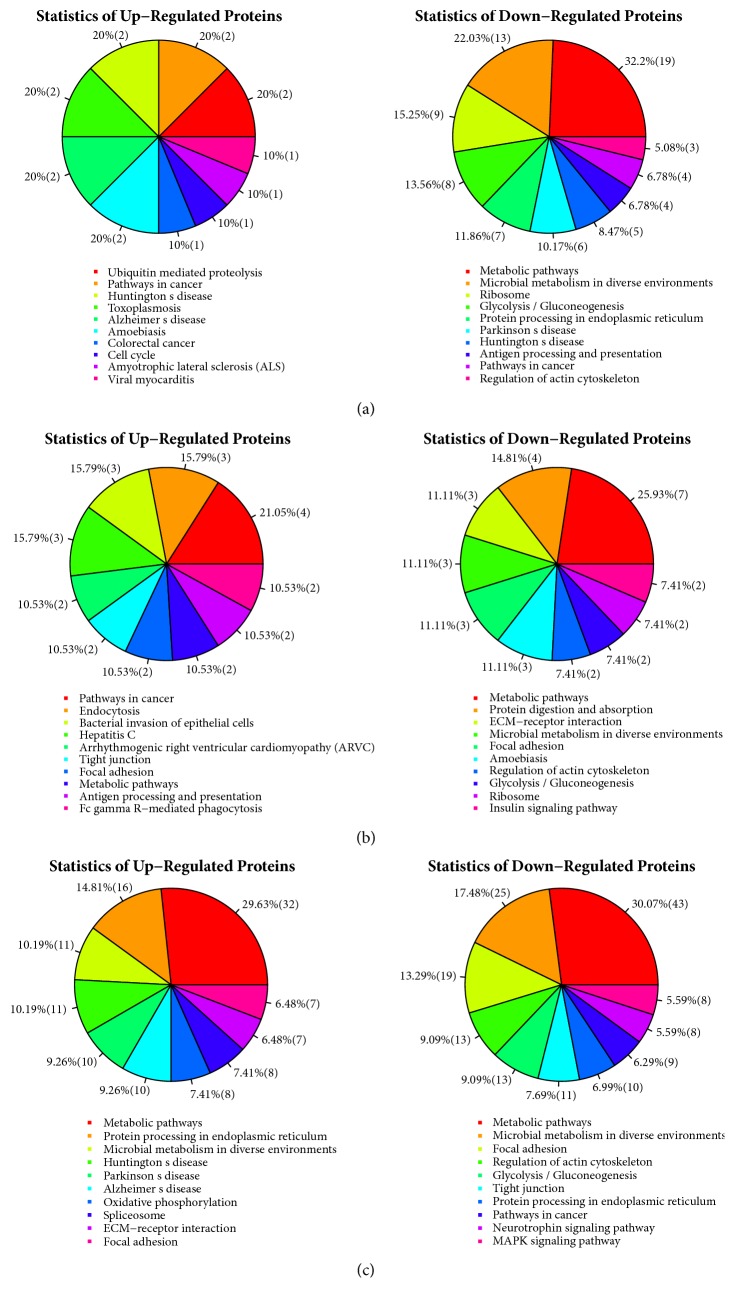
Analysis of the KEGG pathway of the differently expressed proteins at 12 hpi (a), 24 hpi (b), and 42 hpi (c).

The top 10 pathways based on the number of differentially expressed proteins in the DTMUV-infected DEFs at 12, 24, and 42 hpi are shown in Figure 4. For example, of the upregulated proteins, ubiquitin mediated proteolysis (2, 20%), pathways in cancer (2, 20%), Huntington's disease (2, 20%), toxoplasmosis (2, 20%), Alzheimer's disease (2, 20%), amoebiasis (2, 20%), colorectal cancer (1, 10%), cell cycle (1, 10%), amyotrophic lateral sclerosis (ALS) (1, 10%), and viral myocarditis (1, 10%) were involved in the DTMUV-infected DEFs at 12 hpi (Figure 4(a)). Of the upregulated proteins, pathways in cancer (4, 21.05%), endocytosis (3, 15.79%), bacterial invasion of epithelial cells (3, 15.79%), hepatitis C (3, 15.79%), arrhythmogenic right ventricular cardiomyopathy (ARVC) (2, 10.53%), tight junction (2, 10.53%), focal adhesion (2, 10.53%), metabolic pathways (2, 10.53%), antigen processing and presentation (2, 10.53%), and Fc gamma R-mediated phagocytosis (2, 10.53%) were involved in the DTMUV-infected DEFs at 24 hpi (Figure 4(b)). Of the downregulated proteins, metabolic pathways (43, 30.07%), microbial metabolism in diverse environments (25, 17.48%), focal adhesion (19, 13.29%), regulation of actin cytoskeleton (13, 9.09%), glycolysis/gluconeogenesis (13, 9.09%), tight junction (11, 7.69%), protein processing in endoplasmic reticulum (10, 6.99%), pathways in cancer (9, 6.29%), neurotrophin signaling pathway (8, 5.59%), and MAPK signaling pathway (8, 5.59%) were involved in the DTMUV-infected DEFs at 42 hpi (Figure 4(c)).

### 3.5. Analysis of the Identified Proteins at the Transcriptional Level

**Figure 5:**
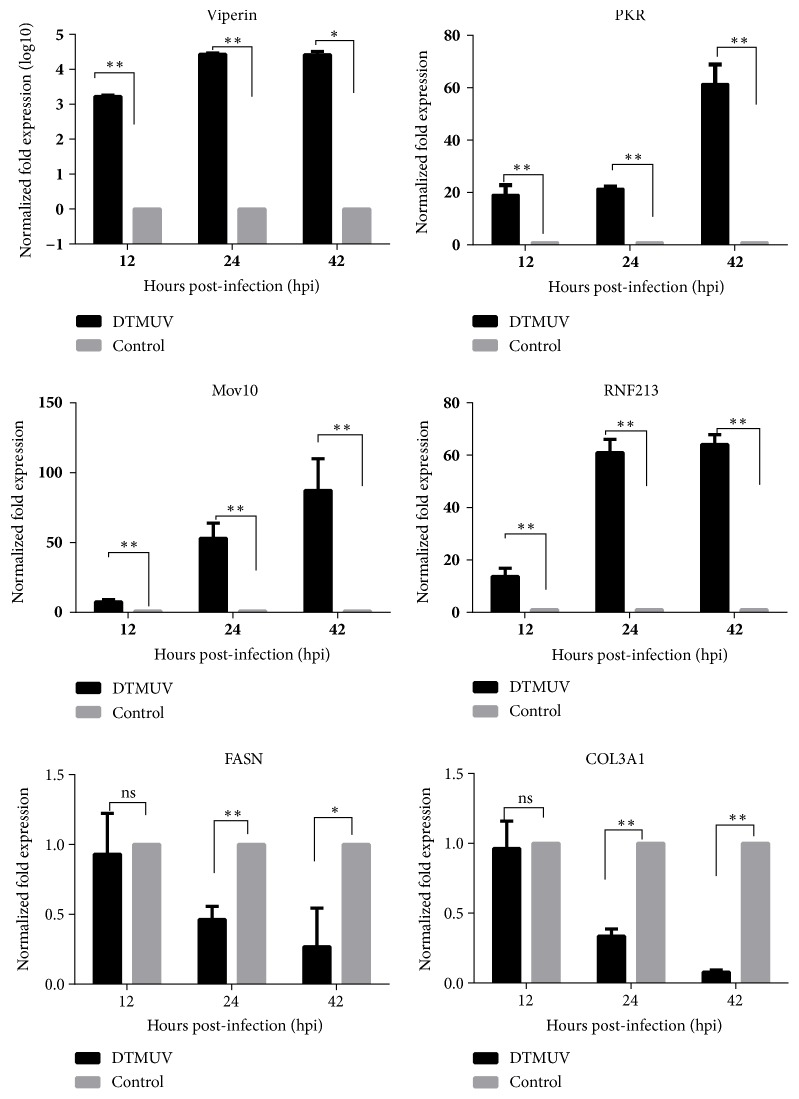
Transcriptional profiles of the differentially expressed proteins in DTMUV-infected DEFs. Error bars represent the standard error of the mean from three independent experiments. *∗*, p <0.05, significantly different; *∗∗*, p <0.01; ns, no significant difference.

The transcriptional alterations in 6 selected proteins were measured by relative quantitative real-time PCR. The results showed that the expression of viperin, double-stranded RNA-dependent protein kinase (PKR), mov10 RISC complex RNA helicase (Mov10), and RING finger protein 213 (RNF213) were upregulated, whereas fatty acid synthase (FASN) and collagen alpha-1(III) chain (COL3A1) were downregulated (Figure 5). The overall real-time PCR results generally matched the iTRAQ data (SI Table 1).

## 4. Discussion

ITRAQ LC-MS/MS is a powerful tool with high sensitivity and quantitation accuracy for proteomic analysis that has been widely applied in many studies [22, 28]. Here, iTRAQ LC-MS/MS was applied to analyze the differential protein expression profiles of duck source DEFs infected with DTMUV at various time points. In this study, a total of 116, 76, and 339 differentially expressed proteins were identified at 12, 24, and 42 hpi based on a fold change >1.5 or <0.67 and p value less than 0.05. The differentially expressed proteins regarding cellular responses were mainly associated with binding, catalytic activity, cellular processes, biological regulation, metabolic processes, response to stimulus, immune system processes, and cell parts. Alterations in the expression of a protein may be owing to a change in its mRNA level. In this study, real-time PCR results were generally in accordance with the proteomic analysis. In addition, some degree of disagreement was observed between these two analyses regarding the upregulated expression of the proteins PKR and viperin at 12 hpi. The protein level of PKR and viperin determined by iTRAQ LC-MS/MS were upregulated with a fold change >1.5, but p value was not less than 0.05 (SI Table 1), which did not correlate with the transcriptional level of these proteins with a significant upregulation (p < 0.05) at 12 hpi. There could be several reasons for this. Gene expression is divided into two levels of transcription and translation, namely, mRNA level and protein level. The time and site of transcription and translation of eukaryotic gene expression were spatiotemporal. Secondly, posttranscriptional mechanisms including protein translation, posttranslational modification, and degradation may influence the level of a protein present in a given cell or tissue [29]. So different regulation mechanisms acting on both the synthesized mRNA and the synthesized protein may account for the amount of the two molecules differentially.

### 4.1. Response to Stimulus and Immune-Associated Proteins

The innate immune system acts as the body's first line of defense against virus infection. The activation of the antiviral innate immune response depends on the pattern-recognition receptors (PRRs) [30]. The PRR family is grouped into the membrane-bound Toll-like receptors (TLRs), C-type lectin receptors (CTLs), retinoid acid-inducible gene-Ι (RIG-I)-like receptors (RLRs), nucleotide binding oligomerization domain (NOD)-like receptors (NLRs), and absent-in-melanoma (AIM)-like receptors (ALRs) [31]. In this study, some of the differentially expressed proteins induced by DTMUV infection in DEFs were involved in the TLR, RLR, NLR, and MAPK signaling pathways, such as cap methyltransferase 1 (CMTR1), DEAD-box helicase 3 X-linked (DDX3X), heat shock protein 90 beta family member 1 (HSP90B1), and ELKS/RAB6-interacting/CAST family member 1 (ERC1). It has been suggested that the TLR, RLR, and NLR signaling pathways play important roles in host cell responses to Flavivirus [32–37].

The expression of some proteins involved in the immune response was altered following DTMUV infection such as interferon stimulated genes (ISGs). In this study, our data indicated that the expression of IFN-induced protein 35 (IFI35), IFIT5, and Mx proteins were significantly upregulated in DTMUV-infected DEFs. IFI35 (also known as IFP35) is a member of the ISGs and can be induced by interferon [38]. IFI35 is a leucine zipper protein and plays an important role in modulating virus infection, innate immune, and inflammatory responses by interacting with various host and viral proteins such as bovine Tas (BTas) regulatory protein of bovine foamy virus [39]. In contrast to the classical role of ISGs in antagonizing virus infections, studies have also shown that IFI35 functions as a negative regulator of RIG-I-mediated antiviral signaling in vesicular stomatitis virus (VSV) infection [40]. In addition, IFI35 can enhance inflammation following H5N1 influenza virus (IAV) infection by increasing proinflammatory cytokine production [41]. However, the possible effects of IFI35 during DTMUV infection need to be explored in future studies.

The IFIT protein family is responsible for nucleic acid sensing during virus infections [42]. The IFITs are evolutionarily conserved, whereas the number of IFIT genes is different between species [43]. Of the IFITs, only IFIT5 has been detected in birds, which was found recently, and knowledge of its function is still obscure. The IFIT5 locus in chicken possesses antiviral activities against negative-sense single-stranded RNA viruses, such as Newcastle disease virus (NDV) [44]. In the present study, the upregulated expression of IFIT5 was induced following DTMUV infection in DEFs; however, its role in the antiviral process and immune regulation requires further study.

### 4.2. Alterations of Metabolism-Associated Proteins

Viral replication requires energy. It has been reported that virus infection can dramatically modify cellular metabolism in a cell. Virus-induced metabolism can increase available energy for virus replication and virion production [45]. In this study, some proteins involved in metabolic processes were found to be differentially expressed in the DTMUV-infected DEFs. Upregulated expressions of the energy metabolism proteins such as UMP-CMP kinase 2, ATP synthase, cytochrome P450 family 51 subfamily A member 1 (CYP51A1), and very long-chain specific acyl-CoA dehydrogenase (VLCAD) were observed in the DTMUV-infected DEFs. Moreover, we also identified some differentially expressed proteins involved in metabolism, including transketolase (TKT), S-(hydroxymethyl) glutathione dehydrogenase, fatty acid synthase, adenosylhomocysteinase (AHCY), and glucose-6-phosphate isomerase (GPI), which were downregulated in DTMUV–infected DEFs.

UMP-CMP kinase belongs to the nucleoside monophosphate (NMP) kinase family and is a known pyrimidine nucleoside monophosphate kinase that phosphorylates CMP, UMP, dCMP, and dUMP [46]. It has been shown that UMP-CMP kinase expression was upregulated during various viral infection [47, 48]. Moreover, UMP-CMP kinase has been reported to be essential for the viability of* Bacillus subtilis* and* Streptococcus pneumoniae* [49]. ATP synthase is one of the most highly conserved enzymes and plays a central role in the synthesis of ATP in all living organisms, and it was originally described from the inner membrane of mitochondria and chloroplast thylakoid membranes [50]. It was also found that the ATP synthase *β* subunit is present on the cell surface where it may serve as a cell membrane receptor [51]. On the other hand, ATP synthase has been identified as a virus-interaction protein capable of mediating the entry of virus into host cells and acts as a factor that mediates human immunodeficiency virus-1 (HIV-1) transfer between antigen-presenting cells and CD4^+^ target cells [52–55]. The rapid replication of DTMUV in host cells will consume abundant host ATP and impact other energy-dependent biological functions of the host cells. Therefore, we propose that the increased amount of ATP synthase and UMP-CMP kinase may be beneficial for DTMUV infection. CYP51A1 is a lanosterol 14*α*-demethylase involved in cholesterol biosynthesis and is present in all biological kingdoms [56]. It has been reported that silence of the expression of CYP51A1 significantly decreased astrovirus replication and particle assembly [57]. VLCAD is a mitochondrial fatty acid oxidation enzyme that is responsible for the rate-limiting step in catabolism of long-chain fatty acids [58]. A previous study indicated that VLCAD deficiency mice show enhanced sensitivity to influenza virus infection due to bioenergetic starvation [59]. Thus, it will be interesting to further investigate the precise role of UMP-CMP kinase, ATP synthase, CYP51A1, and VLCAD in DTMUV infection process.

TKT is a ubiquitous enzyme in cellular carbon metabolism that catalyzes the reversible transfer between ketoses and aldoses as part of the pentose phosphate pathway. This shunt permits cells a flexible adaptation to different metabolic needs as the pentose phosphate pathway produces nicotinamide adenine dinucleotide phosphate (NADPH) for reductive biosynthetic pathways of cholesterol and fatty acids [60]. Furthermore, a previous study showed that TKT had the potential importance and functional involvement in* Rhodopseudomonas palustris* growth [61]. S-(hydroxymethyl) glutathione dehydrogenase is involved in NO metabolism that plays crucial roles in defense responses during host-pathogen interactions. S-(hydroxymethyl) glutathione dehydrogenase is required for conidiation and contributes to virulence in* Magnaporthe oryzae *[62]. Fatty acid synthase (FASN) is a key enzyme in the fatty acid biosynthesis pathway that catalyzes the synthesis of palmitate from acetyl coenzyme A (acetyl-CoA) and malonyl-CoA [63]. The expression of FASN has been reported to be upregulated by the hepatitis C virus core protein, NS2, NS4B, NS5B, and the hepatitis B virus large surface protein by activation of the FASN promoter and plays a role in viral replication [64–66]. FASN inhibitors can also inhibit human cytomegalovirus and IAV replication by modulating the membrane composition for virus budding or protein modifications [67]. Moreover, dengue virus infection promotes lipid biogenesis to benefit virus replication [68]. Therefore, fatty acid synthesis has been identified as a requirement for many virus replications. In the present study, FASN was downregulated following DTMUV infection, which was inconsistent with the reports mentioned above. Since we hypothesize that FASN may play other roles in DTMUV infection, AHCY plays a key role in the control of methylations via regulation of the intracellular concentration of adenosylhomocysteine, which is a competitive inhibitor of S-adenosyl-L-methionine-dependent methyl transferase reactions. AHCY also regulates blood levels of homocysteine, which appear to be risk factors for some diseases [69, 70]. Moreover, AHCY is a target for antiviral drugs, such as 6'-fluoro-3-deazaneplanocin, 6'-isoneplanocin, and 3, 7-dideazaneplanocin [71–73]. GPI as a glycolytic enzyme in glycolysis catalyzes the reversible isomerization of glucose-6-phosphate to fructose-6-hosphate [74]. Besides its role in the glycolytic pathway, mammalian GPI also functions as a tumor-secreted cytokine and an angiogenic factor that stimulates endothelial cell motility. Moreover, GPI is also a neurotrophic factor for spinal and sensory neurons [75]. Furthermore, GPI is the key enzyme which catalyzes the key steps in the glycolysis during white spot syndrome virus (WSSV) infection. Nevertheless, the exploration of exact function of these metabolism-associated proteins during DTMUV infection is an important future study.

### 4.3. Extracellular Matrix (ECM)-Receptor Interaction-Associated Proteins

Collagens are a large family that are primary components of the ECM of metazoa and play an important role in tissue repair, cell migration, cancer, angiogenesis, tissue morphogenesis, and tissue scaffolding. Collagens are also associated with some diseases, such as Alport syndrome, certain arterial aneurysms, Bethlem myopathy and Ullrich muscular dystrophy, Ehlers-Danlos syndrome, and Kniest dysplasia [76]. Ross River virus infection can be inhibited by collagen IV [77]. On the other hand, breakdown of collagen IV and collagen I may facilitate dissemination of* Histophilus somni* infection [78]. Moreover, HIV and hepatitis B virus can promote the expression of type I and IV collagen, respectively, and could be involved in the pathogenesis of virus-associated diseases [79, 80]. In the present study, the upregulated expressions of collagen VI and the downregulation of collagen I, collagen III, and collagen V were induced by DTMUV infection in DEFs. Whether the different types of collagens have different functions in DTMUV infection is not clear.

### 4.4. Neuropathogenicity-Associated Proteins

DTMUV belongs to the genus* Flavivirus*, which also includes West Nile virus, Japanese encephalitis virus (JEV), Yellow fever virus (YFV), and Dengue virus (DENV). Flaviviruses, such as JEV, YFV, and DENV, can cause a pathogenicity of the nervous system [81–83]. Ducks infected by DTMUV have also shown neural symptoms, such as paralysis. Besides flaviviruses, highly-pathogenic avian influenza (HPAI) H5N1 and Newcastle disease (ND) viruses can also cause classic central nervous system dysfunction in poultry and migratory birds. Balasubramaniam* et al*. 2012 [84] have identified twenty-three genes, such as heat shock 70 kDa protein 2 (HSPA2), septin, heat shock 60 kDa protein 1 (HSPD1), and beta-2 microglobulin differentially expressed in chicken brain tissues during infections with HPAI H5N1 and NDV. On the other hand, Gupta* et al*. 2011 [85] have indicated that plectin, lectin, and phosphatidylethanolamine binding protein were differentially expressed during infection with JEV in mice brain. In this study, the differentially expressed proteins heat shock 70 kDa protein 2 (HSPA2), septin, heat shock 60 kDa protein 1 (HSPD1), beta-2 microglobulin, plectin, lectin, and phosphatidylethanolamine binding protein were also identified. Thus, whether these differentially altered proteins are relevant to duck paralysis seen when infected with DTMUV needs to be further studied.

## 5. Conclusions

In summary, the differentially expressed proteins were identified in DTMUV-infected DEFs through iTRAQ analysis. Moreover, DTMUV infection-associated pathways and proteins are described and discussed on the basis of the bioinformatics analysis. Although the roles of the proteins that were identified in this study were not studied, it is likely that all or some of them are involved in host-virus interactions. Therefore, our analysis of the DEFs responses to DTMUV infection provides useful information for a better understanding of the pathogenesis of DTMUV as well as other flaviviruses.

## Figures and Tables

**Table 1:** Primers in this study.

Primers	Sequence (5'→3')	Usage
DTMUV-F	GATAAAGAGGACGATTGATGG	Amplification of DTMUV gene
DTMUV-R	TTCCGCTTATTCAGTCCGT	
V-F	CAGTGATGAAGAATTTGAGC	Amplification of viperin gene
V-R	CTTTCCGTCCATTTCTACAG	
PKR-F	GGCCGTCAATATTTACAG	Amplification of PKR gene
PKR-R	CACGGTGACATAATCAAG	
Mov-F	CTGCAAGGAGAAGGGCGGCTAC	Amplification of Mov10 gene
Mov-R	CCTGAAGGACGGCCCGTGAAAC	
Ring-F	AAATTGGCATGGGATGAGTTAG	Amplification of RNF213 gene
Ring-R	TGCTATATCCTTCTGCGATG	
FASN-F	GCCAACAGGATTTCTTACTTC	Amplification of FASN gene
FASN-R	TGTCCATTACGAATTGCCTTAT	
C-F	TTCCTGGATTACCGGGTCA	Amplification of COL3A1 gene
C-R	GGTTGGCCTGGTGATCCGTTTG	
A-F	CAAAGCCAACAGAGAGAAG	Amplification of *β*-actin gene
A-R	CAGAGTCCATCACAATACCAG	

## Data Availability

The data used to support the findings of this study are included within the article.

## Supplementary Materials

Supplementary Materials Supplementary Table 1: list of significant and differentially expressed proteins identified in DEFs infected with DTMUV.
