# Supplementary material for: ITRAQ-Based Quantitative Proteomics Reveals the Proteome Profiles of Primary Duck Embryo Fibroblast Cells Infected with Duck Tembusu Virus
Source: Biomed Res Int. 2019 Jan 27;2019:1582709. doi: 10.1155/2019/1582709 (PMC6369498; doi:10.1155/2019/1582709)
Supplement: Supplementary Materials — Supplementary Table 1: list of significant and differentially expressed proteins identified in DEFs infected with DTMUV. [file 1582709.f1.pdf]

SI TABLE 1: List of significant and differentially expressed proteins identified in DEFs infected with DTMUV[a]

Accession	Name	Protein Length	%Cov (95)	Fold change in expression / p-value		
				12 hpi	24 hpi	42 hpi
U3J5Y3	Spectrin alpha, non-erythrocytic 1	2477	53.25	0.759/0.1499	0.597/3.19E-08	0.192/0
R0KBE2	Fatty acid synthase	2513	52.69	0.614/0.143	0.603/7.22E-08	0.530/2.89E-08
R0LN18	Spectrin beta chain, brain 1	2364	51.95	0.912/0.057	1.236/0.886	0.244/0
R0JC10	Collagen alpha-1(XII) chain	3085	40.23	0.759/0.361	0.492/3.01E-07	2.377/1.43E-09
U3IJ39	AHNAK nucleoprotein	5537	56.75	0.511/6.08E-11	0.570/8.10E-14	0.073/0
U3ID88	Collagen type VI alpha 3 chain	3136	36.70	0.904/0.819	0.955/0.321	2.109/4.00E-13
U3IIB9	Myosin heavy chain 9	1907	50.03	0.879/0.133	1.499/0.549	1.706/2.54E-06
R0LH70	Fibronectin	2431	44.34	1.486/0.0001	0.879/0.403	2.655/2.53E-12
U3IN65	Plectin	4196	22.71	0.973/0.772	1.236/0.142	0.263/0
U3I6C2	Glutamyl-prolyl-tRNA synthetase	1607	49.47	0.614/0.001	1.028/0.177	0.492/7.34E-07
U3ISA8	Pre-mRNA processing factor 8	2248	30.92	1.096/0.764	1.202/0.904	2.051/0.0002
U3J4X1	Heat shock protein 90 beta family member 1	771	62.13	0.421/0.006	1.722/0.161	2.858/1.07E-12
U3ISP9	Eukaryotic translation elongation factor 2	826	60.41	0.511/0.0002	1.096/0.047	0.398/1.07E-05
R0JSM9	Heat shock protein HSP 90-alpha	732	54.51	0.305/2.77E-08	0.839/0.162	0.5755/0.004
U3J925	ATP-citrate synthase	1101	51.59	0.461/0.007	1.236/0.716	0.525/7.91E-07
R0L1Y3	60 kDa heat shock protein, mitochondrial	573	79.23	0.581/0.001	1.028/0.416	1.837/0.0001
U3IW58	Collagen type I alpha 2 chain	1363	62.36	1.086/0.588	0.555/0.003	0.283/0.0004
U3I992	Talin 2	2544	29.44	1.107/0.767	1.393/0.599	0.350/0.002
U3IR26	Vinculin	1082	49.45	1.019/0.506	0.738/0.094	0.316/1.98E-05
U3I6R1	Annexin	658	51.22	0.316/0.011	0.643/0.0003	0.698/0.041
R0L244	Supervillin	2206	27.61	0.973/0.926	0.904/0.118	0.240/6.70E-07
U3IX93	High density lipoprotein binding protein	1267	44.36	0.631/0.015	0.946/0.320	0.340/1.28E-05
R0L527	Filamin-B	1099	60.42	0.731/0.029	0.887/0.299	0.597/0.004
R0L4K0	Heat shock cognate 71 kDa protein	647	57.03	0.488/0.0002	1.445/0.893	0.570/0.006
R0JMK8	Aminopeptidase	835	47.43	0.724/0.012	0.929/0.194	0.515/0.0003
U3IUB7	Lamin B2	549	59.56	0.705/0.003	1.528/0.280	0.209/8.53E-08
U3IFD3	Myosin heavy chain 11	1979	34.77	0.847/0.026	1.180/0.399	1.820/8.45E-05
U3I640	Heat shock protein family A (Hsp70) member 5	612	57.84	0.679/0.0004	1.905/0.053	2.377/4.21E-09
U3IR52	Alpha-enolase	434	82.03	0.377/0.002	1.096/0.241	0.247/3.30E-12
U3I0F9	Pyruvate kinase	539	70.69	0.535/0.024	0.938/0.040	0.370/4.34E-05

SI TABLE 1: continued

Accession	Name	Protein Length	%Cov (95)	Fold change in expression / p-value		
				12 hpi	24 hpi	42 hpi
R0M765	Elongation factor 1-alpha	462	74.68	0.118/2.88E-05	0.718/0.188	1.486/0.029
U3J5N5	Prolyl endopeptidase	717	50.91	0.879/0.560	0.824/0.034	0.661/0.025
Q7ZYV1	Sodium/potassium-transporting ATPase subunit alpha	1023	35.97	0.520/0.030	1.318/0.707	1.191/0.343
U3J3Z2	Chaperonin containing TCP1 subunit 7	543	62.80	0.497/0.001	0.673/0.038	0.839/0.420
U3IES1	Annexin	340	77.94	0.555/0.005	0.955/0.021	0.794/0.015
R0L497	Cullin-associated NEDD8-dissociated protein 1	1210	32.98	1.148/0.982	0.832/0.041	0.649/0.026
U3IPR3	Lamin A/C	643	52.41	0.964/0.363	1.629/0.002	0.787/0.361
U3J091	Kinectin 1	1349	28.54	0.847/0.274	0.964/0.116	0.291/5.00E-06
U3J4J3	Acetyl-CoA acetyltransferase 1	407	71.01	0.429/0.067	1.294/0.224	2.270/0.0003
U3J9R9	AP-2 complex subunit alpha	936	39.74	0.920/0.858	0.920/0.771	0.483/0.008
U3I4I9	Actinin alpha 1	662	54.38	1.127/0.745	0.817/0.023	0.238/7.46E-06
U3IB14	DnaJ heat shock protein family (Hsp40) member C13	2236	19.81	1.076/0.700	1.180/0.602	1.528/0.003
U3J3X9	5-aminoimidazole-4-carboxamide ribonucleotide formyltransferase / IMP cyclohydrolase	597	66.67	0.809/0.130	1.086/0.839	0.560/0.006
U3J0G1	Chaperonin containing TCP1 subunit 2	535	56.82	0.325/0.002	0.4831/0.004	0.802/0.432
U3IF77	T-complex 1	535	63.55	0.802/0.341	0.637/0.045	0.667/0.547
R0LZB0	Protein disulfide-isomerase	365	68.22	0.219/3.30E-05	1/0.315	1.191/0.076
U3IMD1	Catenin alpha 1	905	42.98	0.759/0.075	0.871/0.137	0.363/0.002
R0KBX6	Protein disulfide-isomerase	615	36.1	0.363/7.34E-05	0.679/0.027	1.786/3.87E-06
U3IZA5	ATP-dependent 6-phosphofructokinase	756	44.18	1.225/0.536	1.236/0.894	1.528/0.013
T2HPU5	Chaperonin containing TCP1, subunit 5 (Epsilon)	399	72.68	0.479/0.026	0.679/0.012	0.809/0.905
U3IEG9	Far upstream element binding protein 1	602	56.31	0.731/0.026	1.191/0.569	0.275/0.027
U3ILF5	Phosphoglycerate kinase	416	74.76	0.360/0.003	1.660/0.805	0.337/0.001
U3I3S5	Nicotinamide nucleotide transhydrogenase	1090	34.5	0.655/0.008	0.863/0.419	1.854/0.001
U3IM50	Septin 9	570	51.93	0.592/0.007	1.294/0.487	0.661/0.017
U3IQ39	Heterogeneous nuclear ribonucleoprotein A3	380	47.89	0.597/0.018	1.570/0.970	0.879/0.718
U3J1L1	Glyceraldehyde-3-phosphate dehydrogenase	333	62.76	0.560/0.018	0.973/0.406	0.964/0.415
U3IIX3	Rab GDP dissociation inhibitor	365	77.26	1.096/0.313	0.855/0.116	0.409/0.001

SI TABLE 1: continued

Accession	Name	Protein Length	%Cov (95)	Fold change in expression / p-value		
				12 hpi	24 hpi	42 hpi
R0LCU1	Coatomer subunit gamma	857	41.19	0.705/0.580	0.625/0.009	0.679/0.0444
R0JWN6	Heterogeneous nuclear ribonucleoprotein M	666	38.74	0.920/0.169	2.070/0.098	2.208/0.001
U3I9L4	Dolichyl-diphosphooligosaccharide-protein glycosyltransferase subunit 1	609	52.05	0.501/0.367	1.047/0.030	1.754/0.002
U3IRX8	SEC31 homolog A, COPII coat complex component	1215	24.28	0.982/0.831	0.982/0.040	0.540/0.021
R0JJW5	Collagen alpha-2(VI) chain	1020	26.18	0.887/0.575	1.047/0.553	1.706/0.001
U3J698	Transketolase	594	49.49	0.497/0.046	0.661/0.004	0.655/0.007
R0LQ61	T-complex protein 1 subunit zeta	464	71.55	0.363/0.008	0.766/0.521	0.904/0.105
U3IE28	Heterochromatin protein 1 binding protein 3	559	38.28	0.575/0.002	1.393/0.309	1.459/0.003
U3IGC1	SEC24 homolog C, COPII coat complex component	1106	28.21	0.855/0.741	0.679/0.053	0.643/0.040
R0KME4	Putative pre-mRNA-splicing factor ATP-dependent RNA helicase DHX15	740	35.949999	0.991/0.960	1.127/0.865	1.738/0.009
U3IEK7	Hypoxia up-regulated 1	973	31.96	1.028/0.905	1.542/0.486	2.228/0.0003
U3IXU2	Heterogeneous nuclear ribonucleoprotein U	547	42.96	0.787/0.023	1.169/0.448	3.076/4.87E-09
U3ILC4	Tyrosine 3-monooxygenase/tryptophan 5-monooxygenase activation protein theta	246	71.95	0.991/0.582	0.637/0.429	0.477/0.011
R0JIB7	Myosin-Ib	1136	21.65	1.236/0.127	1.067/0.888	1.722/0.010
U3I2D1	Ubiquinol-cytochrome c reductase core protein 1	461	52.71	0.946/0.827	0.787/0.585	1.77/0.003
U3ILQ7	DNA topoisomerase I	747	29.72	0.625/0.003	1.259/0.768	0.938/0.381
R0LR18	DNA helicase	749	37.38	1/0.790	1.086/0.575	2.655/0.002
U3IX64	Testin LIM domain protein	423	60.76	0.619/0.007	0.912/0.355	0.649/0.035
U3IDM0	Tyrosine--tRNA ligase	524	44.08	0.991/0.470	0.515/8.55E-05	0.685/0.009
U3IEX4	Adducin 1	736	41.44	1.038/0.953	0.920/0.741	0.185/3.24E-05
U3ICM6	Gelsolin	781	40.72	0.483/0.0002	0.871/0.071	0.380/0.0005
U3I342	Ubiquinol-cytochrome c reductase core protein 2	457	54.05	0.766/0.0228	0.973/0.869	1.690/0.002
U3IUR2	Procollagen-lysine, 2-oxoglutarate 5-dioxygenase 2	727	37.14	1.028/0.650	1.047/0.850	2.032/0.002
U3I7Q0	Heterogeneous nuclear ribonucleoprotein R	636	44.65	0.506/0.006	1.191/0.564	0.711/0.944
U3IFH4	6-phosphogluconate dehydrogenase, decarboxylating	466	48.07	0.575/0.219	0.930/0.163	0.244/1.02E-05

SI TABLE 1: continued

Accession	Name	Protein Length	%Cov (95)	Fold change in expression / p-value 12 hpi	24 hpi	42 hpi
U3I593	Receptor for activated C kinase 1	281	81.49	1/0.553	0.429/0.001	0.525/0.050
U3IIH9	Proteasome 26S subunit, non-ATPase 12	420	57.86	0.938/0.607	0.794/0.335	1.570/0.005
U3I5M4	Mannose receptor C type 2	1451	19.50	0.912/0.971	0.661/0.004	0.353/1.45E-05
U3J597	Isocitrate dehydrogenase [NADP]	415	51.57	0.461/0.002	0.738/0.059	0.299/1.97E-05
U3IN22	Nucleoporin 155	1350	22.52	1.019/0.685	1.159/0.643	1.786/0.002
U3J849	Dedicator of cytokinesis 7	2060	16.60	1.009/0.508	1.644/0.016	1.318/0.007
R0JQR6	Tyrosine-protein kinase-like 7	865	28.9	0.752/0.360	0.802/0.224	0.328/0.0001
R0M3E5	Band 4.1-like protein 2	992	26.71	0.904/0.142	0.912/0.315	0.592/0.025
U3IIL1	Annexin	341	61.58	0.661/0.124	0.904/0.011	0.213/0.0003
R0KW24	40S ribosomal protein S4	236	63.14	0.540/0.167	0.839/0.189	1.888/0.039
U3IZI5	ARP3 actin related protein 3 homolog	418	56.22	0.955/0.844	0.718/0.056	0.497/0.001
R0JFS9	Inosine-5'-monophosphate dehydrogenase	436	51.83	0.614/0.067	0.406/0.001	0.731/0.311
U3ISN7	Laminin subunit beta 1	1719	16.4	1.706/0.037	1.271/0.066	1.096/0.082
U3IV94	Procollagen-lysine,2-oxoglutarate 5-dioxygenase 1	730	37.40	1.236/0.463	1.076/0.804	1.644/0.008
U3IZ48	Lysine--tRNA ligase	554	38.63	1.009/0.422	1.127/0.544	0.643/0.010
R0JKK6	Collagen alpha-1(VI) chain	1008	30.46	0.759/0.162	1.459/0.663	2.333/0.008
R0JMH7	Alpha-actinin-2	897	31.77	1.202/0.632	0.673/0.079	0.242/7.18E-05
U3J7Y9	40S ribosomal protein SA	320	63.44	1.009/0.428	1.343/0.825	0.283/0.041
U3IRV6	Karyopherin subunit beta 1	875	29.49	0.724/0.947	0.637/0.009	0.325/0.004
S4SM24	Eukaryotic initiation factor 4A-III	352	52.27	0.964/0.793	0.912/0.290	1.600/0.004
U3ICG6	Proteasome 26S subunit, non-ATPase 3	389	42.16	0.904/0.357	0.655/0.040	1.514/0.009
U3I8D8	Triosephosphate isomerase	211	87.20	0.492/0.031	1.445/0.529	0.157/1.18E-07
R0JH14	Glutamate dehydrogenase 1, mitochondrial	410	57.560003	1.076/0.994	1.057/0.760	1.528/0.018
U3IQW9	G protein subunit alpha i2	316	58.230001	0.679/0.445	0.929/0.622	2.355/0.011
U3J8L3	Solute carrier family 25 member 13	681	34.36	1.028/0.787	1.038/0.950	1.644/0.038
U3J680	Caldesmon 1	544	36.76	0.738/0.010	1.047/0.886	0.093/4.28E-07
U3IE74	L-lactate dehydrogenase	336	48.21	0.685/0.178	0.550/0.004	0.177/1.08E-06
U3IEQ8	Sjogren syndrome antigen B	423	36.64	0.520/0.002	1.180/0.566	0.964/0.676
R0LRH2	60S ribosomal protein L4	297	40.74	0.752/0.127	0.724/0.312	1.977/0.034
R0L6A3	Bifunctional 3'-phosphoadenosine 5'-phosphosulfate synthetase 1	604	37.09	0.991/0.815	1.096/0.596	1.923/0.011
U3J188	DnaJ homolog subfamily C member 10	795	25.28	1.038/0.563	1.294/0.875	1.514/0.008
R0LJ39	Peroxiredoxin-1	201	66.67	0.535/0.010	1/0.202	1.271/0.214

SI TABLE 1: continued

Accession	Name	Protein Length	%Cov (95)	Fold change in expression / p-value		
				12 hpi	24 hpi	42 hpi
R0L8K1	Core histone macro-H2A.1	372	54.84	0.863/0.295	0.895/0.339	1.675/0.014
U3IAY7	Acyl-CoA dehydrogenase long chain	428	37.85	1.038/0.744	1.871/0.392	1.706/0.013
R0L597	Leukotriene A(4) hydrolase	559	29.16	0.920/0.427	0.855/0.167	0.429/0.001
U3I4I4	Fermitin family member 2	684	36.99	0.738/0.165	1.086/0.742	0.535/0.014
U3J830	Peroxidasin	1419	17.62	1.117/0.350	0.856/0.243	0.506/0.010
U3IBB9	Nuclear autoantigenic sperm protein	719	30.46	0.738/0.112	1.159/0.363	0.425/0.007
R0LC54	DNA repair protein RAD50	1312	14.86	1.117/0.252	1.225/0.558	1.542/0.011
A0A0B5JQU7	Viperin	363	42.70	3.565/0.059	32.509/2.42E-08	28.840/1.27E-07
U3IC85	Ribosomal protein L7a	270	44.07	0.492/0.017	0.731/0.137	1.499/0.032
U3IIW5	Tyrosine 3-monooxygenase/tryptophan 5-monooxygenase activation protein epsilon	234	73.08	0.832/0.048	0.698/8.22E-05	0.360/0.006
R0LH42	ATP-binding cassette sub-family E member 1	610	38.03	0.824/0.583	0.575/0.010	0.759/0.147
R0M4W3	A-kinase anchor protein 12	959	21.38	0.773/0.128	1.019/0.710	0.121/2.36E-05
R0LRM7	Destrin	165	79.39	0.137/0.012	0.360/0.004	0.535/0.100
R0LLK8	Decorin	357	56.02	0.745/0.296	0.800/0.437	2.333/0.002
U3II57	Cytochrome P450 family 51 subfamily A member 1	483	34.58	0.824/0.099	1/0.995	2.208/0.002
U3J3V1	Electron transfer flavoprotein dehydrogenase	622	35.53	0.759/0.656	1.213/0.671	1.675/0.003
U3IUJ4	Ubiquitin like modifier activating enzyme 2	591	43.65	0.661/0.025	1.282/0.419	1.009/0.794
R0KHM3	Interferon-induced protein with tetratricopeptide repeats 5	480	36.25	5.395/0.029	40.551/6.78E-08	51.050/3.34E-09
U3J083	DNA helicase	808	25.87	1/0.795	0.938/0.689	1.770/0.012
U3INE4	Carnosine dipeptidase 2	478	40.38	0.794/0.444	0.912/0.185	0.308/1.35E-06
U3J4F7	Proline and arginine rich end leucine rich repeat protein	319	55.49	1.057/0.543	0.724/0.053	1.977/0.004
U3IWQ6	Adenylyl cyclase-associated protein	475	34.53	0.787/0.332	0.938/0.749	0.474/0.001
U3J5E2	S-(hydroxymethyl)glutathione dehydrogenase	376	36.17	0.525/0.004	0.575/0.005	0.619/0.032
U3J9R8	Alpha-1,4 glucan phosphorylase	836	32.54	0.839/0.772	1.067/0.721	0.540/0.012
U3INI1	DEAD-box helicase 5	602	51.33	1.148/0.500	1.472/0.782	1.837/0.0277
U3J4E5	Ribosomal protein S3	247	57.09	1.380/0.605	0.724/0.077	1.738/0.044
R0M2V6	Ubiquitin-like modifier-activating enzyme 6	1003	19.04	1.528/0.049	0.887/0.809	1.306/0.173
U3IR67	Hydroxysteroid 17-beta dehydrogenase 4	740	24.32	1.905/0.057	1.057/0.708	1.888/0.039

SI TABLE 1: continued

Accession	Name	Protein Length	%Cov (95)	Fold change in expression / p-value 12 hpi	24 hpi	42 hpi
U3ICC7	Coronin	480	30.63	0.738/0.339	1.159/0.832	0.592/0.049
U3IUC4	Actin-related protein 2/3 complex subunit	370	46.76	0.855/0.431	0.990/0.307	0.501/0.008
R0L313	Neurogenic locus notch-like protein 2	2412	11.4	1.656/0.014	1/0.338	0.895/0.867
U3ILE9	Apoptosis inhibitor 5	522	24.71	0.787/0.502	0.787/0.198	0.575/0.049
U3ISF4	Succinyl-CoA:3-ketoacid-coenzyme A transferase	516	35.66	0.483/0.149	0.479/0.010	1.294/0.274
U3ILN2	Ribosomal protein S2	234	58.97	0.363/0.012	0.470/0.008	1.419/0.124
U3IWX3	Sorting nexin	401	36.16	0.608/0.033	1.294/0.474	0.982/0.762
R0K3R7	Melanoma inhibitory activity protein 3	1913	10.61	0.592/0.014	0.787/0.304	1.117/0.421
U3INM7	Carnitine palmitoyltransferase 2	655	26.11	0.863/0.156	0.964/0.216	1.923/0.012
R0LBH9	Peptidyl-prolyl cis-trans isomerase	134	82.84	0.581/0.003	1.368/0.769	1.406/0.121
U3IFW5	Voltage dependent anion channel 3	296	60.81	0.453/0.040	0.731/0.093	1.472/0.073
U3J1N9	GTP-binding nuclear protein Ran	216	46.76	0.350/0.006	0.603/0.627	1.675/0.010
U3IRI9	Collagen type V alpha 1 chain	1841	14.94	0.586/0.742	0.488/0.091	0.275/0.0007
U3J2Z0	Heterogeneous nuclear ribonucleoprotein A/B	263	41.83	0.445/0.033	1.318/0.409	0.625/0.087
U3J175	ATP synthase peripheral stalk-membrane subunit b	228	47.81	0.895/0.157	0.759/0.035	1.600/0.033
R0KGG1	BAT2 domain-containing protein 1	2752	11.34	0.525/0.092	1.614/0.221	0.288/0.006
U3IN94	Keratin 19	413	36.56	0.711/0.255	0.429/0.014	1.820/0.042
R0JMV5	Bleomycin hydrolase	420	34.52	0.377/0.016	0.780/0.202	0.457/0.020
R0M3M9	Collagen alpha-1(III) chain	1453	22.71	0.752/0.982	0.515/0.009	0.085/8.56E-05
U3J9U8	DNA helicase	823	16.4	1.159/0.577	0.832/0.374	1.629/0.0135
U3IU67	Calponin	331	46.22	0.832/0.111	0.592/0.023	0.510/0.111
U3J383	Phosphoglucomutase 1	566	29.51	0.817/0.756	0.692/0.047	0.575/0.019
U3ITQ7	Protein disulfide isomerase family A member 6	444	33.78	0.643/0.121	2.582/0.776	1.644/0.038
U3I8X7	Malic enzyme	583	29.50	1.057/0.973	0.964/0.911	1.556/0.040
U3I6I6	Atlastin GTPase 1	558	28.32	0.802/0.981	0.6199/0.588	1.820/0.024
R0M0E4	Microtubule-associated protein RP/EB family member 1	226	50.88	0.453/0.004	0.7319/0.202	0.540/0.004
R0KXC4	Protein DJ-1	189	79.37	0.809/0.216	0.766/0.489	0.268/0.0002
U3IB74	Protein O-fucosyltransferase 1	306	57.52	0.863/0.205	1.038/0.574	1.888/0.016
U3ISV5	Centrosomal protein 170	1579	10.07	1.528/0.047	1.380/0.114	0.955/0.749
U3J8S6	MutS homolog 6	1280	11.25	0.863/0.151	1.393/0.238	1.644/0.014
U3J4Z3	Nucleosome assembly protein 1 like 4	354	35.59	1.117/0.793	1.086/0.746	0.474/0.009
R0LKT3	Receptor protein-tyrosine kinase	1075	17.67	1.159/0.541	0.887/0.356	0.501/0.002
R0LNH9	Collagen alpha-2(V) chain	1464	21.99	0.946/0.976	1.180/0.876	0.421/0.048

SI TABLE 1: continued

Accession	Name	Protein Length	%Cov (95)	Fold change in expression / p-value 12 hpi	24 hpi	42 hpi
U3I321	2',3'-cyclic nucleotide 3' phosphodiesterase	422	31.28	1.660/0.384	3.565/0.0003	4.325/6.65E-07
U3I8R9	NADH dehydrogenase [ubiquinone] 1 alpha subcomplex subunit 10, mitochondrial	355	47.61	0.904/0.557	1.170/0.983	1.514/0.038
R0JFQ9	Peroxiredoxin-6	142	77.46	0.592/0.143	1.191/0.706	0.263/0.0006
R0JK78	Phosphate carrier protein, mitochondrial	309	36.57	0.661/0.316	1.019/0.923	1.629/0.017
U3IVD1	RB binding protein 4, chromatin remodeling factor	425	44.24	0.586/0.429	0.832/0.864	0.251/0.022
U3ILP7	Peptidase D	497	31.59	0.973/0.554	0.871/0.634	0.236/4.28E-05
U3J8D3	High mobility group box 3	180	45.56	0.603/0.036	0.929/0.946	0.711/0.176
U3HYT7	Cellular retinoic acid binding protein 1	135	65.93	0.619/0.038	0.625/0.023	0.305/0.001
R4HH66	Desmin	302	57.28	1.076/0.952	0.929/0.740	0.270/0.001
U3IQT7	UTP--glucose-1-phosphate uridylyltransferase	509	27.31	0.817/0.031	1.086/0.765	0.432/0.006
R0LR13	ELKS/RAB6-interacting/CAST family member 1	1117	13.16	1.067/0.677	1.247/0.088	0.614/0.015
U3IH78	Dolichyl-diphosphooligosaccharid e--protein glycosyltransferase 48 kDa subunit	377	33.42	1.009/0.777	1.127/0.786	2.270/0.038
A0A0M4R WG0	Zinc finger CCCH-type antiviral protein 1	717	20.36	2.291/0.002	3.221/7.41E-0 5	2.291/0.001
U3J057	Phosphatidylethanolamine binding protein 1	151	64.90	1.057/0.375	1.247/0.605	0.185/0.0003
U3IBU3	Prohibitin 2	231	43.72	0.759/0.086	0.787/0.583	2.032/0.020
R0LIL9	ATP synthase subunit O, mitochondrial	184	60.87	0.319/0.012	0.990/0.434	1.995/0.005
U3J894	Proteasome subunit alpha type	255	50.20	0.614/0.077	1.294/0.622	0.387/0.026
R0J6Z5	60S acidic ribosomal protein P0	137	67.88	0.425/0.019	0.661/0.690	0.649/0.807
R0LQ12	23 kDa cortical cytoskeleton-associated protein	244	53.28	0.082/0.009	0.506/0.101	0.437/0.005
U3IZC4	Glutaredoxin 3	334	35.63	0.938/0.482	0.832/0.712	0.631/0.020
U3I6H8	Laminin subunit gamma 1	1544	10.23	1.236/0.850	1.660/0.033	1.722/0.016
U3ILU0	40S ribosomal protein S12	132	74.24	0.655/0.008	0.832/0.792	0.673/0.032
R0K0M8	Cofilin-2	166	74.1	0.673/0.154	0.649/0.039	0.492/0.033
R0L513	Eukaryotic translation initiation factor 3 subunit I	325	44.62	0.724/0.203	1.019/0.452	0.597/0.038
U3IXX2	SAC1 like phosphatidylinositide phosphatase	589	22.24	1.191/0.795	0.955/0.238	1.585/0.039

SI TABLE 1: continued

Accession	Name	Protein Length	%Cov (95)	Fold change in expression / p-value		
				12 hpi	24 hpi	42 hpi
U3J5C4	Squalene epoxidase	478	26.15	0.946/0.615	1.057/0.817	1.905/0.021
R0KZE1	Glucocorticoid receptor DNA-binding factor 1	1425	9.05	1.406/0.119	2.014/0.022	1.380/0.471
R0JD86	Cathepsin B	340	32.35	0.955/0.447	0.824/0.934	0.207/0.003
R0JS60	Proteasome endopeptidase complex	246	54.47	0.718/0.343	1.259/0.628	0.308/0.015
U3IHK6	Glyoxalase domain containing 4	300	43.33	0.766/0.150	1.271/0.896	0.288/0.002
U3IQE2	Eukaryotic translation termination factor 1	409	36.92	0.904/0.816	0.955/0.128	0.331/0.028
R0L2K9	ATP-dependent RNA helicase DDX42	940	15.74	1.306/0.230	1.556/0.232	0.406/0.012
U3IEH8	Acetyl-CoA acetyltransferase 2	336	48.81	1.138/0.607	0.887/0.831	0.441/0.009
U3J9N9	Microtubule associated protein RP/EB family member 2	288	40.28	0.643/0.013	0.895/0.865	0.895/0.550
U3J0H8	Elastin microfibril interfacer 2	1035	13.14	1.445/0.563	1.923/0.019	1.754/0.058
U3J0N2	NEDD8-activating enzyme E1 regulatory subunit	536	24.63	1.754/0.042	1.236/0.613	0.773/0.458
R0JXM5	Malate dehydrogenase	334	33.53	0.817/0.211	0.946/0.053	0.217/0.021
R0KAH1	Protein SEC13-like protein	304	41.45	0.787/0.237	0.871/0.559	0.603/0.043
R0JYN0	Prostaglandin F2 receptor negative regulator	878	13.1	1.202/0.438	1.282/0.140	1.585/0.031
U3IDM4	Raftlin family member 2	432	4.86	0.603/0.005	17.219/0.020	1.905/0.007
U3IDM1	Small nuclear ribonucleoprotein polypeptide A'	234	29.06	1.096/0.440	0.929/0.997	0.603/0.034
U3HYT9	Ribosomal protein L23a	156	43.59	0.483/0.004	0.501/0.066	1.169/0.212
R0KET6	Glutathione S-transferase 3	223	35.43	0.780/0.491	1.009/0.259	0.244/0.002
U3IN21	BH3 interacting domain death agonist	193	67.36	0.895/0.672	2.704/0.182	1.754/0.01
R0JNU8	Acidic leucine-rich nuclear phosphoprotein 32 family member A	199	35.18	0.673/0.052	0.686/0.049	0.437/0.030
R0JBT4	RING finger protein 213	4165	3.99	2.754/0.007	3.597/0.0002	2.965/5.94E-07
U3ITT8	Proteasome endopeptidase complex	222	56.31	0.946/0.245	0.692/0.379	0.525/0.010
U3IWZ8	Phosphomannomutase	234	39.32	0.879/0.699	0.879/0.672	0.550/0.021
U3IL75	Phosphoglucomutase 2	611	15.22	0.817/0.869	0.938/0.590	0.299/0.020
R0LM55	Translin	176	37.5	0.855/0.794	1.009/0.665	0.380/0.037
U3IQA5	Cystathionine beta-synthase	568	18.84	1.096/0.797	1.306/0.392	0.501/0.027
U3J3H8	RAB3 GTPase activating protein catalytic subunit 1	986	12.98	1.722/0.026	1.169/0.776	0.511/0.180
U3J679	Nucleoporin 160	1335	9.74	1.660/0.329	1.585/0.201	1.77/0.024
U3ITG1	Pre-mRNA processing factor 39	591	17.6	0.863/0.665	1.169/0.662	2.355/0.006
U3IR35	Stathmin	149	32.21	0.698/0.366	0.643/0.302	0.291/0.034
U3J7N6	Sperm associated antigen 9	1207	10.77	1.076/0.408	1.445/0.096	0.474/0.001

SI TABLE 1: continued

Accession	Name	Protein Length	%Cov (95)	Fold change in expression / p-value		
				12 hpi	24 hpi	42 hpi
R0K108	Double-stranded RNA-binding protein Staufen-like protein 1	712	19.24	1.009/0.551	0.929/0.831	0.540/0.029
U3I946	Arp2/3 complex 34 kDa subunit	300	34.33	0.685/0.097	0.698/0.211	0.614/0.047
R0JP63	Farnesyl pyrophosphate synthetase	258	24.81	0.817/0.864	0.809/0.906	0.545/0.045
U3IQF4	NADH-cytochrome b5 reductase	300	47.67	1.236/0.638	0.540/0.029	1.77/0.123
R0K221	Huntingtin-interacting protein 1	973	11.82	1.542/0.088	1.690/0.040	1.318/0.304
U3I6U1	Rho GDP dissociation inhibitor beta	206	69.42	0.773/0.458	0.802/0.061	0.377/0.002
U3INF1	Mitochondrial calcium uniporter	301	32.56	0.839/0.859	1.271/0.450	2.489/0.007
U3J6G2	Tropomyosin 3	252	58.33	0.929/0.069	0.895/0.458	0.497/0.004
U3IPL5	Golgin B1	3201	6.59	1.170/0.167	1.213/0.296	0.586/0.016
R0LZW3	Thymocyte nuclear protein 1	228	38.60	0.738//0.931	1.028/0.606	0.163/0.024
U3ITC1	STT3B, catalytic subunit of the oligosaccharyltransferase complex	776	10.7	1.514/0.816	1.127/0.969	2.356/0.003
U3I7S4	Zyxin	581	23.06	1.019/0.621	1.107/0.576	0.360/0.016
U3J8B7	RUN and FYVE domain containing 3	600	14	1.259/0.411	1.432/0.544	2.109/0.039
R0LI37	FKBP12-rapamycin complex-associated protein	2516	5.92	1.096/0.528	2.312/0.003	0.855/0.436
R0LJZ9	UMP-CMP kinase 2, mitochondrial	149	53.69	1.393/0.240	16.444/0.018	18.535/0.001
U3IQV1	Glycylpeptide N-tetradecanoyltransferase	454	20.93	0.752/0.585	0.920/0.747	0.466/0.043
R0K4R2	Cell division control protein 42-like protein	192	34.38	0.310/0.0005	0.555/0.208	0.912/0.524
U3I676	Branched-chain-amino-acid aminotransferase	381	18.11	1.191/0.711	1.380/0.465	0.441/0.038
R0LR53	Catenin alpha-2	553	26.04	0.773/0.997	1.923/0.021	0.649/0.279
U3I8M2	Interferon induced protein 35	300	32.67	5.297/0.002	16.904/5.06E-05	12.023/6.56E-05
U3ITE5	Lectin, mannose binding 2	276	22.1	0.692/0.149	0.773/0.354	1.888/0.029
R0M464	Eukaryotic translation initiation factor 3 subunit J	171	57.31	0.871/0.685	1.057/0.590	0.242/0.007
R0M884	Glutathione S-transferase	222	33.78	0.824/0.448	0.685/0.140	0.108/0.001
R0JDK1	Hemoglobin subunit pi	142	49.30	0.350/0.004	0.055/0.0004	0.356/0.035
U3ID57	Aminopeptidase	949	6.96	1.419/0.235	1.770/0.1290	1.722/0.014
U3J0D3	Epsin 2	664	12.50	1.169/0.248	0.824/0.187	0.520/0.049
U3INZ8	SH3 domain-binding glutamic acid-rich-like protein	114	68.42	0.745/0.459	1.180/0.509	0.138/0.008
U3J5E9	Osteoclast stimulating factor 1	207	38.65	0.929/0.589	1.107/0.854	0.421/0.020
U3I6H4	Lactoylglutathione lyase	185	32.43	0.711/0.421	0.773/0.530	0.337/0.006
U3I632	Calcium/calmodulin dependent protein kinase kinase 2	484	16.74	1.959/0.027	1.343/0.2993	1.318/0.240

SI TABLE 1: continued

Accession	Name	Protein Length	%Cov (95)	Fold change in expression / p-value		
				12 hpi	24 hpi	42 hpi
U3IQH4	Protein-L-isoaspartate O-methyltransferase	227	48.90	1.009/0.575	0.991/0.533	0.661/0.036
U3IHG9	Ribosomal protein S20	118	49.15	0.265/0.189	0.391/0.209	0.530/0.049
U3IEL6	Stomatin	282	26.24	1.786/0.023	1.306/0.243	0.787/0.680
R0LDU8	VPS10 domain-containing receptor SorCS2	920	8.80	0.581/0.200	0.847/0.270	0.603/0.049
U3J4D8	Prefoldin subunit 3	194	32.47	0.955/0.649	0.964/0.479	0.432/0.021
U3IFS6	Thioredoxin domain containing 17	75	56.00	0.731/0.961	1.191/0.945	0.421/0.029
R0K419	Heme oxygenase 1	297	22.90	0.938/0.308	0.787/0.293	0.322/0.006
U3I0C5	Lymphocyte cytosolic protein 1	626	18.21	1.754/0.238	1.318/0.685	0.238/0.018
Q1XHT3	Beta-2-microglobulin	96	68.75	3.373/0.135	13.804/0.003	9.036/0.003
U3IA01	Hematopoietic prostaglandin D synthase	202	23.27	0.631/0.604	0.545/0.020	0.766/0.570
R0J8D8	Cartilage intermediate layer protein 2	420	15.95	7.727/0.009	23.550/0.001	19.055/0.005
A0A0A1CT T9	Yes-associated protein 1	415	19.28	1.057/0.947	1.159/0.573	0.254/0.047
U3IS83	Arginyl aminopeptidase	447	13.42	1.057/0.997	0.766/0.118	0.441/0.033
U3IZD8	Solute carrier family 25 member 22	277	16.25	1.138/0.894	1.180/0.699	2.630/0.014
U3IQD4	Leucine zipper and CTNNBIP1 domain containing	190	27.89	1.406/0.956	1.047/0.732	0.373/0.041
U3HZM9	Glutathione reductase	493	14.00	1.271/0.916	1.629/0.043	0.673/0.682
R0L0F4	Double-stranded RNA-specific adenosine deaminase	935	5.78	1.514/0.347	3.342/0.036	1.445/0.289
R0LJJ2	Poly [ADP-ribose] polymerase	1803	3.44	1.076/0.462	2.938/0.026	2.535/0.731
U3J929	SUMO-conjugating enzyme	162	22.84	0.661/0.049	0.738/0.091	0.855/0.464
R0LB59	Protein phosphatase 1G	522	14.18	0.180/0.003	0.614/0.080	1.500/0.887
U3IYB8	Cell division cycle 37	233	20.17	0.511/0.608	0.413/0.036	0.575/0.258
R0LPW1	Huntingtin	3092	2.10	1.977/0.050	1.585/0.106	1.380/0.509
U3IAD3	Tumor protein, translationally-controlled 1	138	23.91	0.470/0.045	0.667/0.378	0.938/0.933
U3IQE3	Glycerol-3-phosphate dehydrogenase [NAD(+)]	350	16.57	0.847/0.580	0.685/0.692	0.242/0.023
R0KBG0	Metalloproteinase inhibitor 3	212	13.21	1.259/0.787	0.705/0.659	3.048/0.025
R0K8T8	Sterile alpha motif domain-containing protein 9-like	1537	3.97	1.236/0.030	1.644/0.053	3.105/0.004
U3INW9	Connective tissue growth factor	267	11.99	0.597/0.153	1.380/0.391	6.546/0.029
A0A0U2V8 A4	Alpha D globin subunit	141	37.59	0.384/0.330	0.217/0.008	0.787/0.126
U3IBX8	FRAS1 related extracellular matrix protein 2	3044	1.58	1.432/0.055	1.528/0.0230	1.282/0.323

SI TABLE 1: continued

Accession	Name	Protein Length	%Cov (95)	Fold change in expression / p-value		
				12 hpi	24 hpi	42 hpi
U3IGS4	AP complex subunit beta	952	44.01	0.679/0.041	0.879/0.188	0.550/0.027
U3HZJ9	Ribosome binding protein 1	954	40.99	1.086/0.409	1.343/0.513	0.203/2.46E-12
R0L6N6	Protein disulfide-isomerase	425	74.82	0.356/6.15E-05	1.282/0.468	1.722/9.50E-05
R0JMZ7	T-complex protein 1 subunit theta	528	71.0	0.667/0.106	0.525/0.001	0.855/0.847
R0LE95	Alpha-1,4 glucan phosphorylase	828	49.03	1.107/0.581	0.938/0.162	0.384/0
R0JUU9	Cytoskeleton-associated protein 4	444	70.72	0.738/0.049	1.282/0.864	1.675/0
U3I0A3	Keratin 7	492	66.87	0.461/0.004	0.441/0	2.148/7.99E-05
U3I935	Moesin	578	59.86	0.570/0.005	1.271/0.884	0.215/3.01E-07
U3IC15	Aconitate hydratase, mitochondrial	786	51.27	0.560/0.086	0.752/0.258	1.923/0.001
U3IGY5	Aspartate aminotransferase	427	78.69	0.466/0.010	1.542/0.896	1.871/0.001
R0KF12	Nuclear pore complex protein Nup93	819	29.06	1.432/0.055	0.824/0.916	1.542/0
U3J0T9	Insulin like growth factor 2 mRNA binding protein 3	584	46.75	0.667/0.054	1.306/0.494	2.208/0.040
R0LMN3	DNA helicase	890	31.57	1.009/0.479	1.096/0.947	1.706/0.013
U3IUB6	Elongation factor Tu GTP binding domain containing 2	972	33.64	0.991/0.653	0.991/0.721	1.629/0.017
R0KDL4	26S proteasome non-ATPase regulatory subunit 1	935	31.66	0.912/0.888	0.794/0.169	1.614/0.013
U3I939	Fructose-bisphosphate aldolase	335	68.66	0.887/0.066	3.076/0.265	0.479/7.02E-05
R0JQ38	Tenascin	2172	16.44	0.964/0.702	0.955/0.119	3.192/1.56E-05
U3IA60	Malate dehydrogenase	338	77.22	0.655/0.073	0.794/0.220	1.570/0.007
R0KN24	Xaa-Pro aminopeptidase 1	624	54.33	0.817/0.272	0.685/0.0153	0.575/0.025
R0LVN5	Echinoderm microtubule-associated protein-like 4	970	24.85	0.847/0.479	0.809/0.440	0.113/6.34E-06
U3J8P0	Mov10 RISC complex RNA helicase	924	27.60	1.600/0.042	11.803/2.37E-06	15.996/4.05E-09
R0JGG2	Hydroxymethylglutaryl-CoA synthase	525	44	0.488/0.040	0.745/0.011	0.738/0.325
U3IXM0	Peptidylprolyl isomerase	422	48.82	0.614/0.009	0.773/0.011	0.441/0.001
R0LHZ1	3-ketoacyl-CoA thiolase, mitochondrial	397	59.7	0.895/0.455	0.964/0.452	1.722/0.024
U3IRP3	Glucose-6-phosphate isomerase	560	47.68	0.387/0.066	0.625/0.395	0.265/0
U3IGP4	Aspartate beta-hydroxylase	735	36.05	0.802/0.108	0.991/0.608	1.599/0.005
U3IYF3	Voltage dependent anion channel 2	294	59.18	0.337/0	0.773/0.807	1.419/0.146
U3J9A1	WD repeat domain 1	636	41.35	0.773/0.071	0.855/0.311	0.530/0

SI TABLE 1: continued

Accession	Name	Protein Length	%Cov (95)	Fold change in expression / p-value		
				12 hpi	24 hpi	42 hpi
A0A1P8LK38	2',5'-oligoadenylate synthetase-like protein	503	48.11	10.568/9.63E-05	24.434/6.78E-07	56.494/3.13E-10
R0M0V9	Multifunctional protein ADE2	420	47.62	0.863/0.386	0.718/0.126	0.575/0.009
U3IUG2	Plastin 3	640	38.91	0.673/0.114	0.773/0.044	0.347/0
R0LPZ6	Thioredoxin domain-containing protein 5	348	74.14	0.597/0.373	0.802/0.274	1.660/0.013
U3J8P3	Ribosomal protein L5	297	48.82	0.506/0.006	0.780/0.364	0.655/0.104
R0LD68	Adenosylhomocysteinase	433	43.65	0.497/0.011	0.667/0.009	0.278/0
U3II47	Aldo-keto reductase family 1 member A1	328	68.60	0.685/0.032	0.824/0.635	0.525/0.009
U3J4Q2	Capping actin protein of muscle Z-line beta subunit	272	66.18	0.608/0.035	1.086/0.043	0.387/0.004
U3I4C0	Tyrosine 3-monooxygenase/tryptophan 5-monooxygenase activation protein zeta	247	72.06	0.310/0.128	0.679/0.509	0.380/0.003
U3IR48	Dihydrolipoyl dehydrogenase	505	36.63	0.373/0.003	0.920/0.409	1.419/0.139
U3ISI9	Transaldolase	305	52.46	0.432/0	0.802/0.357	0.302/0
R0JLG5	Calnexin	591	25.21	0.337/0.021	0.461/0.183	2.291/0
R0L8I2	Prohibitin	275	73.45	0.982/0.491	0.955/0.534	1.690/0.009
R0LYS8	RuvB-like helicase	409	43.28	1.028/0.798	0.871/0.754	1.820/0.013
R0M1C8	40S ribosomal protein S7	201	61.69	0.229/0.002	0.673/0.742	1.271/0.157
U3IEA5	Cortactin	556	31.65	0.637/0.009	0.991/0.928	0.199/0.007
U3IB92	Ribophorin II	576	31.60	0.586/0.271	0.863/0.141	2.070/0
U3IUS7	NSFL1 cofactor	358	43.3	1.037/0.226	1.047/0.226	0.099/1.94E-06
U3IEZ5	Polypyrimidine tract binding protein 1	557	45.24	0.718/0.287	0.809/0.025	0.545/0.006
P33238	Interferon-induced GTP-binding protein Mx	721	25.38	2.249/0.221	12.474/1.28E-05	24.434/2.68E-10
P13743	L-lactate dehydrogenase B chain	333	48.65	0.614/0.007	0.711/0.002	0.221/0
U3I4Q4	Nucleoside diphosphate kinase	269	56.51	0.479/0.010	1.047/0.623	0.150/1.82E-05
U3I535	Aldo-keto reductase family 1 member D1	326	56.13	0.946/0.695	0.973/0.554	0.164/0
U3I4P2	SAMM50 sorting and assembly machinery component	466	33.26	1.660/0.039	1.282/0.932	1.499/0.016
R0LL58	RNA-binding protein 12	880	18.75	0.904/0.946	0.649/0.036	1.159/0.809
R0LRZ1	Thrombospondin-1	1148	15.68	1.180/0.219	0.871/0.873	4.487/3.72E-07
U3J9S1	Proteasome subunit alpha type	261	57.47	0.809/0.716	0.817/0.226	0.520/0.027

SI TABLE 1: continued

Accession	Name	Protein Length	%Cov (95)	Fold change in expression / p-value 12 hpi	24 hpi	42 hpi
R0L8K4	Transforming growth factor-beta-induced protein ig-h3	621	22.87	1.086/0.582	0.718/0.180	3.221/0
U3IZX9	Proteasome endopeptidase complex	216	56.94	0.731/0.054	0.780/0.079	0.316/0.006
U3J840	60S ribosomal protein L6	277	40.43	0.643/0.163	2.831/0.555	1.770/0.035
R0LJH4	Selenide, water dikinase 1	393	44.78	0.839/0.927	0.938/0.488	0.466/0.020
U3I004	Chloride intracellular channel protein	253	51.38	0.608/0.047	0.661/0.149	0.655/0.336
U3IK89	ATP synthase, H+ transporting, mitochondrial F1 complex, gamma polypeptide 1	474	20.89	0.745/0.096	1.236/0.924	1.959/0.021
R0KYG8	HEAT repeat-containing protein 1	2114	7.10	1.247/0.358	1.019/0.177	1.905/0.014
R0M006	Alpha-aminoadipic semialdehyde synthase, mitochondrial	926	14.36	0.973/0.713	1.770/0.007	1/0.359
U3IFA9	Vaccinia related kinase 1	413	28.57	0.973/0.843	0.973/0.741	2.051/0.015
U3IDJ5	Reticulon	1024	16.31	0.991/0.703	0.603/0.121	2.051/0.017
A0A172QNP5	Superoxide dismutase [Cu-Zn]	154	85.06	0.817/0.406	0.920/0.254	0.157/0
U3J5F9	Cytochrome c oxidase subunit 5A	150	47.33	0.679/0.188	0.912/0.743	1.675/0.019
U3IDU6	Ribosomal protein	214	37.38	0.247/0.008	0.409/0.007	1.282/0.223
R0JSN7	Histone-lysine N-methyltransferase	593	19.73	0.946/0.885	0.608/0.029	1.0186/0.346
U3J173	TSR1, ribosome maturation factor	738	16.4	1.096/0.459	1.472/0.197	1.923/0.019
R0JKW0	Cytochrome c	107	57.94	0.449/0.018	0.698/0.215	0.156/0
R0JTR9	Proteasome subunit beta type	203	51.23	0.773/0.310	1.067/0.291	0.394/0.008
U3J521	Peptidylprolyl isomerase D	368	27.72	0.643/0.036	0.879/0.986	0.824/0.153
U3J3N7	ENAH, actin regulator	763	14.02	0.619/0.100	0.802/0.664	0.305/0.007
R0L4L6	LIM and SH3 domain protein 1	221	32.58	1.107/0.416	2.606/0.040	0.305/0.002
U3IJ51	Tubulin alpha chain	454	68.94	1.057/0.771	2.377/0.972	2.729/0.017
U3IYK8	RAD23 homolog B, nucleotide excision repair protein	401	20.45	0.973/0.713	1.107/0.659	0.545/0.032
U3IZ93	Cathepsin K	335	39.40	0.847/0.779	0.631/0.280	0.229/0.005
R0KYP5	Hemoglobin subunit rho	147	50.34	0.2911/0.026	0.145/0.007	1.445/0.814
U3ICK7	Phosphoserine aminotransferase	371	23.72	0.794/0.610	1.191/0.811	0.461/0.026

SI TABLE 1: continued

Accession	Name	Protein Length	%Cov (95)	Fold change in expression / p-value 12 hpi	24 hpi	42 hpi
U3IZR4	Paxillin	590	20.85	0.766/0.880	0.794/0.242	0.363/0.035
U3J6D5	Caspase 3	275	30.91	1.644/0.017	1.159/0.771	1.271/0.491
A0A0M4S8A2	Double-stranded RNA-dependent protein kinase	502	10.76	6.982/0.134	32.810/0.002	25.351/0
R0JMN5	Abhydrolase domain-containing protein 12	336	19.35	1.803/0.026	1.770/0.089	1.419/0.069
R0JSB0	UPF0553 protein C9orf64-like protein	227	33.04	1.213/0.296	0.560/0.048	1.047/0.858
R0KTZ1	Polypeptide N-acetylgalactosaminyltransferase	559	10.38	2.014/0.028	0.832/0.165	1.138/0.753
R0K2W7	Glycerol-3-phosphate dehydrogenase, mitochondrial	562	8.185	1.318/0.378	1.138/0.976	2.208/0.029
U3J3R4	Cap methyltransferase 1	832	5.4	1.180/0.556	2.128/0.084	3.020/0.025
R0JVD4	Bullous pemphigoid antigen 1, isoforms 6/9/10	7442	1.384	1.028/0.877	1.977/0.008	1.117/0.547
R0KA42	Rho GDP-dissociation inhibitor 1	204	34.31	0.773/0.827	0.839/0.519	0.370/0.046
Q6I7L3	MHC class I antigen	354	23.16	1.977/0.280	2.992/0.105	5.346/0.048
U3IGA8	Transformation/transcription domain associated protein	3819	2.96	2.99/0.006	1.1169/0.882	3.133/0.006
A0A1L5SM08	MHC class I antigen	353	9.92	1.995/0.269	5.546/0.041	5.012/0.017
U3J6G0	Histone H2A	140	52.14	0.121/0.022	1.644/0.198	0.817/0.8877
R0M3Z3	Myristoylated alanine-rich C-kinase substrate	33	66.67	0.052/0.028	0.078/0.144	0.550/0.656
U3IZ29	Component of oligomeric golgi complex 7	734	3.00	2.014/0.017	0.824/0.457	1.169/0.579
C7ELZ8	Hemoglobin beta A subunit	147	36.73	0.166/0.033	0.466/0.261	0.887/0.730
U3J7G5	Heme binding protein 1	178	14.04	1.057/0.812	0.929/0.926	0.196/0.007
U3IMI2	NOC2 like nucleolar associated transcriptional repressor	684	3.07	0.631/0.007	1.148/0.334	0.938/0.498
U3I008	Uncharacterized protein	2781	0.97	0.938/0.817	0.863/0.553	1.644/0.021
U3J7T0	Uncharacterized protein	1877	34.42	1.086/0.699	1.096/0.666	0.631/0.027
U3IPY8	Uncharacterized protein	1407	47.19	0.766/0.019	0.938/0.027	0.370/0.002
U3ITH8	Uncharacterized protein	2247	30.22	0.545/0.001	0.855/0.090	0.871/0.489
U3IA67	Uncharacterized protein	808	63.49	0.802/0.907	0.887/0.127	0.249/1.45E-07
U3IVL6	Uncharacterized protein	500	60.61	1.570/0.583	1.600/0.588	2.559/0.009
U3IRL7	Uncharacterized protein	471	42.68	1.706/0.213	1.380/0.205	0.172/1.11E-05
U3J9U3	Uncharacterized protein	923	31.31	1.459/0.767	0.221/0.001	0.036/0.002
U3I6B2	Uncharacterized protein	2224	8.27	1.191/0.174	1.432/0.084	0.540/0.016

SI TABLE 1: continued

Accession	Name	Protein	%Cov	Fold change in expression / p-value		
		Length	(95)	12 hpi	24 hpi	42 hpi
U3J090	Uncharacterized protein	450	26.44	1.169/0.896	1.138/0.957	0.205/0.028
U3ILL9	Uncharacterized protein	187	44.39	0.331/0.008	0.839/0.661	1.247/0.511
U3IUN7	Uncharacterized protein	385	28.05	0.483/0.077	1.770/0.941	0.506/0.027
U3I5Z1	Uncharacterized protein	330	45.76	1.057/0.638	0.964/0.826	0.429/0.005
U3IIX9	Uncharacterized protein	968	13.12	1.057/0.518	1.600/0.048	0.780/0.412
U3IWS8	Uncharacterized protein	924	27.06	0.705/0.360	1.271/0.098	1.837/0.033
U3I6K3	Uncharacterized protein	307	54.07	0.887/0.891	0.724/0.381	0.310/0.030
U3J5I9	Uncharacterized protein	146	63.70	0.955/0.485	1.294/0.428	0.457/0.0227
U3J0V8	Uncharacterized protein	580	21.03	2.109/0.111	10.471/9.52E-05	8.710/2.22E-05
U3J1Q5	Uncharacterized protein	227	63.00	0.802/0.463	0.705/0.282	0.520/0.017
U3ID58	Uncharacterized protein	208	55.29	0.824/0.383	0.802/0.023	0.437/0.014
R0LGF6	Uncharacterized protein	918	16.12	1.225/0.803	1.330/0.235	1.738/0.045
U3I731	Uncharacterized protein	1286	10.11	0.904/0.776	0.946/0.634	0.555/0.046
U3IIW2	Uncharacterized protein	444	25.23	0.328/0.348	0.402/0.030	0.492/0.010
U3J662	Uncharacterized protein	163	63.19	0.488/0.103	0.780/0.165	0.310/0.027
U3I2N1	Uncharacterized protein	679	16.64	0.920/0.781	0.973/0.751	1.995/0.042
R0J946	Uncharacterized protein	459	26.36	0.457/0.023	0.592/0.217	0.406/0.024
U3IK46	Uncharacterized protein	149	61.07	0.406/0.401	0.247/0.014	0.215/0.019
U3IE65	Uncharacterized protein	242	36.78	0.787/0.414	0.780/0.683	0.370/0.025
U3IMG4	Uncharacterized protein	97	40.21	0.353/0.036	0.643/0.485	0.258/0.094
U3IRS0	Uncharacterized protein	219	21.00	1.432/0.598	0.488/0.175	2.128/0.046
U3J3R0	Uncharacterized protein	655	58.63	1.096/0.513	1.159/0.306	1.959/0.003
U3J2L5	Uncharacterized protein	1072	37.69	0.840/0.727	0.603/0.002	0.540/0.033
U3I806	Uncharacterized protein	760	49.47	0.643/0.207	0.920/0.357	1.528/0.001
U3I8U0	Uncharacterized protein	543	47.15	1.202/0.762	1.419/0.612	1.570/0.023
U3IM27	Uncharacterized protein	521	50.48	0.982/0.734	1.368/0.490	2.051/1.94E-05
U3ILW3	Uncharacterized protein	916	33.08	1.067/0.593	0.991/0.582	0.530/0.010
U3IQA4	Uncharacterized protein	264	57.95	0.832/0.379	1.644/0.856	3.221/0.001
U3IR89	Uncharacterized protein	402	47.26	0.855/0.524	0.855/0.137	0.497/0
U3I6S1	Uncharacterized protein	474	49.37	0.955/0.737	1.393/0.558	1.871/0.010
U3J947	Uncharacterized protein	673	24.37	0.904/0.718	0.912/0.140	0.511/0
U3J8E6	Uncharacterized protein	277	72.92	0.718/0.355	0.946/0.761	0.437/0.021
U3IP12	Uncharacterized protein	1158	22.37	1.138/0.392	1.282/0.209	0.549/0.001
U3IZV9	Uncharacterized protein	282	27.3	0.863/0.407	1.629/0.244	2.312/0.013
U3INB7	Uncharacterized protein	85	38.82	0.081/0.005	7.311/0.905	0.631/0.355
R0JE94	Uncharacterized protein	106	48.11	0.982/0.003	11.376/0.022	23.768/0.023

[a]Note: % Cov (95) means percent coverage (95%). Proteins with fold change >1.5 or <0.67 and a p-value <0.05 were considered as significantly different expressions.
